# Supplementary material for: Geriatrics as a specialty: Insights from Mexican medical students’ decision-making process
Source: PLoS One. 2025 Feb 14;20(2):e0315096. doi: 10.1371/journal.pone.0315096 (PMC11828400; doi:10.1371/journal.pone.0315096)
Supplement: Attachment — Submitted filename: Response to reviewers 18-11-2024.docx [file pone.0315096.s001.docx]

*Mexico City, November 18^th^, 2024*

Solna Carreon Santos

Editor
PLOS ONE

We are submitting a revised version of our manuscript: “Geriatrics as a Specialty: Insights from Mexican Medical Students' Decision-Making Process” PONE-D-24-29160. According to your letter of November 18^th^, 2024:

*“Please ensure that you include a title page within your main document. You should list all authors and all affiliations as per our author instructions and clearly indicate the corresponding author”.*

According to your instructions:

- The title page was included in the main manuscript.

- The list of authors was modified according to the instructions for authors, institutional positions and academic titles were eliminated and the symbols indicated in the instructions were used.

The corresponding author was specified according to the instructions:

* Corresponding author

alfonso.gutierrezc@incmnsz.mx cirlos@hotmail.com (CGC)

Thank you very much for your quick response and patience.

Kind regards.

Carlos Gutiérrez-Cirlos

Faculty of Medicine, National Autonomous University of Mexico, Mexico City, Mexico.
